# Do traditional medicine-based diets lead to greater weight loss than modern diets in overweight and obese students? A randomized controlled trial

**DOI:** 10.1186/s12906-026-05289-3

**Published:** 2026-02-07

**Authors:** Mohsen Farboud, Elham Haghjoo, Mohammad Mehdi Naghizadeh, Somayeh Abolghasemi, Maryam Moradi, Fatemeh Zarepour, Fatemeh Heidarinejad, Mohammad Ariya

**Affiliations:** 1 Department of Public Health, School of Health, Fasa University of Medical Sciences, Fasa, Iran; 2 Department of Persian Medicine, Fasa University of Medical Sciences, Fasa, Iran; 3 Noncommunicable Diseases Research Center, Fasa University of Medical Sciences, Fasa, Iran; 4 Department of Nutrition, Fasa University of Medical Sciences, Fasa, Iran; 5 School of Public Health, Kerman University of Medical Sciences, Kerman, Iran; 6 School of Health Sciences, Western Sydney University, Campbelltown, Australia

**Keywords:** Obesity, Overweight, Students, Weight loss, Iranian traditional medicine, Modern diets, Dietary interventions

## Abstract

**Background:**

Obesity in adolescents is a critical health concern linked to severe adulthood conditions; therefore, this randomized controlled trial compared the relative effectiveness of a modern dietary approach versus a traditional Iranian medicine-based diet for weight management.

**Methods:**

Following simple randomization (stratified by gender and school type), 93 junior high school students were assigned to either a modern diet (300–500 kcal/day deficit, *n* = 41) or a traditional Iranian diet (qualitative humoral-based, *n* = 52) for 90 days. Dietary adherence was assessed using a 10-point Visual Analog Scale (VAS). Baseline demographic, anthropometric, and unhealthy eating pattern data were recorded.

**Results:**

Over 90 days, the modern diet group achieved a statistically significant decrease in BMI (mean change: -0.31 kg/m²; *P* = 0.032) and waist circumference (*P* < 0.001). Conversely, the traditional Iranian diet group showed a significant increase in weight and BMI (mean change: +0.74 kg/m²; *P* < 0.001). Between-group differences for BMI change were highly significant favoring the modern diet (*P* < 0.001). Dietary adherence and feasibility scores improved in the modern group (*P* = 0.033) but decreased in the traditional group (*P* = 0.001).

**Conclusions:**

These findings suggest that the modern dietary approach was more effective for weight loss in this student population, likely due to better adherence influenced by greater public knowledge and accessibility of information, alongside healthier dietary pattern shifts.

**Trial registration:**

The study is registered with the Iranian Registry of Clinical Trials (IRCT20230610058442N1 dated September 10, 2023).

**Supplementary Information:**

The online version contains supplementary material available at 10.1186/s12906-026-05289-3.

## Introduction

Obesity has emerged as a critical global health concern, particularly in developing nations where prevalence often exceeds that of developed countries [1]. Addressing this issue among adolescents is vital, as excess weight during these formative years is strongly linked to irreversible physical and mental health consequences [2]. Moreover, if left unmanaged, adolescent obesity can lead to long-term, potentially irreversible consequences for both physical and mental health [3]. Therefore, effective management during adolescence is essential to prevent chronic adult conditions, such as type 2 diabetes and cardiovascular disease, while significantly reducing the risk of premature mortality and long-term disability [2, 3].

Research indicates that lifestyle interventions (comprising dietary changes, increased physical activity, and behavioural modification) are the first-line treatment for managing overweight and obesity in adolescents [4, 5]. Among these, structured dietary interventions that focus on modifying eating patterns and controlling caloric intake play a pivotal role [6]. Nutrition professionals often implement these strategies to design age-appropriate, sustainable meal plans for adolescents, aiming to curb the rising prevalence of obesity during this critical developmental stage [6, 7]. Modern dietary plans, often developed by nutrition professionals, are designed not only to support weight loss or maintenance but also to promote healthier eating habits by encouraging the consumption of nutrient-dense foods and reducing the intake of ultra-processed and harmful items [6, 8].

On the other hand, some studies suggest that dietary approaches rooted in traditional medicine may offer greater effectiveness in promoting weight loss compared to certain modern diets. These traditional regimens often emphasize holistic principles, natural ingredients, and long-term balance, which may contribute to better adherence and metabolic outcomes in some individuals [9, 10]. Among various traditional medical systems, Iranian traditional medicine offers particularly comprehensive and influential dietary guidelines, which have been utilized for centuries in managing body weight and promoting overall health [11]. Indeed, Iranian traditional medicine proposes a variety of strategies for the management of overweight and obesity, which, if appropriately implemented, may contribute to mitigating the prevalence of these conditions among school-aged students [12, 13].

Despite a limited number of studies exploring the differences between modern dietary approaches and the recommendations of Iranian traditional medicine for promoting weight loss [12, 14], no research has specifically compared these two dietary patterns among students to date. The existing gap in scientific knowledge lies in the lack of research specifically comparing the effectiveness of calorie-controlled modern protocols with traditional Iranian medicine-based diets in a student population. This comparison is essential to determine whether qualitative traditional principles can achieve comparable outcomes to quantitative modern restrictions. Therefore, this study aims to evaluate and compare the effectiveness of these two distinct dietary patterns in reducing weight among overweight and obese Iranian students.

## Methods

### Study design and participants

This randomized controlled trial (RCT) employed a parallel-group, pre-test–post-test design. The trial was conducted without any deviations from the pre-specified protocol, outcomes, or analysis plan, and with no involvement from students, teachers, or family members of participants in its design, conduct, or reporting. A CONSORT flow diagram illustrating the study procedure is provided in Supplementary Fig. 1. The study population comprised secondary school students (both male and female), aged 13–16 years, as the target population. This age group was selected because these students are at an age where they possess a basic understanding of dietary habits, yet are not under the intense academic pressure experienced by students in higher grades who are preparing for university entrance examinations, pressures that might hinder adherence to dietary interventions. The study was conducted in the city of Fasa, located in Fars Province in southwestern Iran. According to the most recent census data, Fasa has a population of approximately 250,000 residents [15].

Notably, the study protocol followed the principles of the Declaration of Helsinki and received approval from the Ethics Committee of Fasa University of Medical Sciences (Approval Code: IR.FUMS.REC.1402.064). As the participants were minors (aged 13–16 years), written informed consent to participate was obtained from their parents or legal guardians. Additionally, all student participants provided their written assent to participate. Furthermore, the study was registered with the Iranian Registry of Clinical Trials (IRCTID: IRCT20230610058442N1) on 10/09/2023.

### Sampling method and school selection

In this study, sampling was conducted from both Health-Promoting Schools and regular Schools in 2023–2024. Health-Promoting Schools are institutions that adopt a comprehensive approach to enhance the health of students, staff, and families by fostering healthy learning environments, implementing health-oriented policies, and engaging the local community [16].

For the sampling process, schools were identified using the official registry of the Fasa Department of Education. Within each group, one-quarter of the schools were chosen from among the Health-Promoting Schools. In total, four schools met the eligibility criteria and were included. From each selected school, eligible students were randomly chosen to participate in the study. The selected students were then randomly assigned to one of two dietary intervention groups: (1) a modern diet plan tailored to caloric intake and supervised by a nutritionist, or (2) a traditional Iranian medicine-based dietary plan for weight reduction, overseen by a specialist in traditional Iranian medicine. Participants received no concomitant care for the duration of the study.

### Modern diet planning framework by a nutritionist

Dietary planning for adolescents requires a thorough evaluation of their nutritional status, physical growth, and lifestyle habits [6, 17]. In this process, nutritionists begin the assessment by examining anthropometric measurements such as height, weight, and BMI, using age- and sex-specific growth charts to determine the individual’s nutritional condition [6, 18].

Energy needs for adolescents are determined by age, sex, level of physical activity, and their growth spurt stage [6, 17, 19]. Generally, adolescents within this age range require a higher caloric intake than younger children due to their rapid physical development and increased muscle mass [20]. Moderately active boys aged 13 to 16 typically need about 2,400 to 2,800 kilocalories daily, whereas girls of the same age and activity level require approximately 2,000 to 2,200 kilocalories per day [6, 19]. These values can vary based on whether the adolescent leads a sedentary, moderately active, or highly active lifestyle [6, 17]. Notably, the modern diet was designed to induce a moderate energy deficit (approximately 300–500 kcal/day) relative to the participant’s total daily energy expenditure, based on WHO growth standards and standard clinical guidelines for adolescent weight management [6, 18].

The suggested macronutrient distribution for adolescents indicates that 45 to 65% of total energy should be derived from carbohydrates, 10 to 30% from proteins, and 25 to 35% from fats, with a focus on incorporating unsaturated fats [6, 19]. A well-structured dietary plan also should include three main meals and two to three snacks daily to ensure adequate energy and nutrient intake for growth and cognitive function [6, 19]. Breakfast, recognized as a crucial meal, plays a significant role in enhancing concentration and academic performance and should feature protein sources, dairy products, and whole grains [6, 20]. Moreover, the dietary plan should emphasize nutrient-rich whole foods and healthy fats while limiting processed and sugary items to support optimal metabolic and cognitive health [6]. In this study, the dietary plan developed by nutrition specialists is referred to as the “Modern Diet” A sample one-day diet for this group is provided in Supplementary Table 1.

### Traditional Iranian diet planning framework by Iranian traditional medicine practitioner

The dietary planning method in Iranian traditional medicine is rooted in humoral theory, which classifies individuals and foods based on their inherent qualities—hot, cold, moist, and dry—and aims to maintain balance among these temperaments to promote health [12–14, 21]. According to traditional scholars, food exerts significant effects on various organs of the body, and depending on its qualitative temperament and interaction with bodily systems, it can produce either beneficial or harmful outcomes [14, 21]. This approach considers not only the nutritional content of food but also its qualitative effects on the body, digestion, and temperament [12, 13]. These effects are believed to influence not only physical health but also psychological well-being. Therefore, careful selection of food and beverages is considered essential to prevent disturbances in mental and emotional equilibrium [13].

In traditional Iranian medicine, personalized nutrition is emphasized, where dietary recommendations are tailored to an individual’s mizaj (temperament), age, season, and lifestyle [14, 21]. Indeed, Mizaj was assessed clinically by a qualified Persian Medicine specialist using traditional indices from Avicenna’s Canon (e.g., pulse diagnosis, physical signs, symptomatic history). This clinical assessment serves as the gold standard for classification into categories (e.g., warm-dry, cold-moist) [22]. For example, individuals with a warm and dry temperament are advised to consume foods with cold and moist qualities to restore balance [21]. The dietary regimen in traditional Iranian medicine places individuals within a structured and health-oriented framework for a specific period, encouraging disciplined eating habits [14, 21]. This approach to health preservation emphasizes principles such as proper hydration practices, avoidance of incompatible food combinations, sleep hygiene, regular physical activity, emotional regulation (e.g., managing anger and fear), exposure to clean air, and thorough mastication of food [12–14, 21]. Moreover, meal timing and food combinations are also critical components. traditional Iranian medicine recommends consuming the heaviest meal at midday and avoiding incompatible food combinations, such as fish with dairy, which are believed to disrupt digestion and humoral balance [12, 14]. Indeed, the protocol explicitly prohibited ‘incompatible’ food combinations, particularly the co-consumption of fish and yogurt. In Persian Medicine, distinct rules regarding ‘Oral Food’ dictate that foods with similar cold tempers must not be consumed simultaneously. Since both fish and yogurt are classified as having a ‘cold temper,’ their combination is believed to induce mal-temperament (Soo-e-mizaj) [23]. Unlike the modern diet, the traditional Iranian diet was implemented as an *ad libitum* protocol and did not impose a specific quantitative caloric restriction. This methodological decision was made to ensure fidelity to the traditional reference texts, which emphasize qualitative food balance and satiety cues over quantitative energy calculation. Instead, it focused on qualitative changes. In this study, the dietary plan developed by Iranian traditional medicine practitioners is referred to as the ‘Traditional Iranian Diet.’ An example of this plan is provided in the appendix (Supplementary Table 2).

### Demographic and anthropometric measurements

A demographic checklist was used to collect data on participants’ age, gender, history of previous weight loss diets, type of school (health-promoting vs. regular), and family size. Anthropometric measurements—including weight, height, body mass index (BMI), and waist circumference (WC)—were recorded by trained personnel at baseline and subsequently on days 30, 60, and 90 following the intervention. Weight was measured in kilograms using a Seca digital scale (Model 874, Germany) with participants wearing minimal clothing and no shoes. Height was measured in centimetres without shoes, with participants standing upright. BMI was calculated by dividing weight (kg) by height squared (m²).

According to the World Health Organization (WHO), overweight and obesity in adolescents aged 5–19 years are defined using BMI-for-age Z-scores: overweight is classified as a BMI greater than + 1 standard deviation (equivalent to a BMI of 25 kg/m² at age 19), and obesity is defined as a BMI greater than + 2 standard deviations (equivalent to a BMI of 30 kg/m² at age 19) [6, 18].

### Assessment of dietary adherence score, feasibility, and family cooperation

In the present study, Visual Analog Scales (VAS) were employed to assess dietary adherence score, the feasibility score of following dietary patterns, and the level of family cooperation. The VAS is a simple scale ranging from 1 to 10, where 1 indicates “no adherence/feasibility” and 10 indicates “complete adherence/feasibility. Participants were asked to respond to these items on days 30, 60, and 90 following the initiation of the dietary intervention. For each question, students were instructed to select a number between 1 and 10 that best represented their experience. While VAS provides a subjective estimate of compliance, it was selected for its feasibility in this adolescent population; limitations regarding its subjectivity are acknowledged in the discussion.

### Assessment of unhealthy dietary patterns, appetite levels, meal skipping, and physical activity

To evaluate appetite levels, the present study utilized the 8-item Council of Nutrition Appetite Questionnaire (CNAQ). Responses on the CNAQ are scored on a 5-point Likert scale ranging from A to E (A = 1, B = 2, C = 3, D = 4, E = 5), yielding a total score between 8 (minimum) and 40 (maximum) [24]. The psychometric properties of the CNAQ have been validated in the previous study, demonstrating significant correlations with the total score and subdomains of the Appetite, Hunger, and Sensory Perception (AHSP) questionnaire [25]. The CNAQ has shown acceptable internal consistency, with a Cronbach’s alpha of 0.72 [24].

Physical activity was assessed using a brief questionnaire comprising three items that evaluate the frequency, intensity, and duration of physical activity [26]. The product of these three components yields a composite physical activity score, ranging from below 20 (indicative of a sedentary lifestyle) to above 81 (indicative of a very active lifestyle) [26]. This instrument has been validated in a previous study [25].

To assess the effectiveness of the dietary intervention on unhealthy eating patterns, the frequency of consumption of junk food (e.g., chips, puffs), fast food, and soft drinks was recorded weekly. These data were collected both prior to the dietary intervention and again at 90 days post-intervention. Additionally, the frequency of all meal and snack skipping was assessed at baseline and on day 90.

### Inclusion and exclusion criteria

Participants were eligible for this study if they were first secondary school students, aged 13–16 years, and classified as overweight or obese. The exclusion criteria included lack of consent to participate in the study, severe physical or psychological impairments that would prevent the individual from responding to study instruments, and the presence of psychiatric disorders such as psychosis or conditions that impair memory, such as Alzheimer’s disease. Additional exclusion criteria encompassed adherence to special diets (e.g., vegetarianism), extreme physical disabilities that hinder participation, gastrointestinal disorders such as celiac disease, autoimmune diseases such as multiple sclerosis, and various forms of cancer.

### Assessment of adverse effects (Harms)

Throughout the 90-day intervention, we systematically monitored participants for adverse effects. In weekly follow-up sessions, a nutritionist (modern diet) or a Traditional Iranian Medicine specialist (traditional diet) documented any reported issues, such as gastrointestinal discomfort, fatigue, or food intolerance. Each event was classified by severity (mild, moderate, or severe) and assessed for its likely relationship to the dietary intervention. No serious or life-threatening adverse events were reported.

### Sample size calculation

Based on a previous study comparing the effects of a modern diet and traditional Iranian medicine, the maximum reported standard deviation (SD) of BMI was 1.32, and the mean reduction in BMI following the intervention was reported to be 1.62 units [12]. The required sample size was calculated using the following formula:

$$\begin{array}{cc}\mathrm{d}=1&\mathrm{S}=1.32\\\upalpha=0/05&\mathrm{Z}1=1.96\\\upbeta=0.1&\mathrm{Z}2=1.28\end{array}$$  

$$\mathrm{n}=\frac{2{\mathrm{S}}^{2}}{{\mathrm{d}}^{2}}+\left(\mathrm{Z}1+\mathrm{Z}2\right){}^{2}$$

$$\mathrm{n}\approx\:37$$

The estimated sample size for each group was determined to be 37 participants. To account for potential attrition, 40 participants were recruited into each group.

### Blinding and randomization procedures

Eligible students were randomly assigned to the modern diet group or traditional Iranian diet group using simple randomization, stratified by gender and school type (health-promoting vs. regular). The allocation sequence was generated using a computer-based random number generator. Allocation was concealed using sealed, opaque envelopes prepared by an independent researcher not involved in recruitment. This method ensured that neither the participants nor the enrolling researchers could predict the assignment.

### Statistical methods

Independent t-tests were used to compare quantitative variables, while chi-square tests were employed for the comparison of categorical variables. To assess within-group differences over time, repeated measures analysis of variance (RM-ANOVA) and paired t-tests were employed, while the McNemar test was used for categorical variables. Analysis of covariance (ANCOVA) was applied to compare outcomes across groups at different measurement points. All statistical analyses were performed using SPSS software, version 22 (*P* < 0.05). Due to the low-risk nature of the interventions, no interim analyses or stopping rules were planned, and the study was conducted to completion without interruption.

## Results

Ninety-three first-grade junior high school students (48 boys and 45 girls) participated in the study. All of these students completed the intervention, and no participants withdrew from the study. Although the initial recruitment target was 40 participants per group, high volunteer interest and the ethical inclusion of all eligible candidates during the recruitment period resulted in the enrolment and randomization of 93 students. As no participants withdrew (0% attrition), the final sample size (*n* = 93) exceeded the pre-specified minimum, yielding a post-hoc statistical power greater than 90%.

The mean age was 13.56 ± 0.09 years for boys and 13.80 ± 0.1 years for girls (*P* = 0.72). Forty-one students received a modern diet from an expert nutritionist, while 52 students followed a traditional Iranian diet prescribed by an Iranian traditional medicine practitioner. It is important to note that participants were allocated using simple randomization, which naturally resulted in unequal group sizes (*n* = 41 Modern vs. *n* = 52 Traditional). As supported by methodological guidelines [27], this approach prioritizes allocation concealment and unpredictability over numerical equality. Our initial comparison confirmed that this imbalance did not result in significant baseline differences between groups (Table 1).

**Table 1:** Changes in anthropometric measures over 90 days in response to two dietary interventions

Variable	Modern Diet (*n* = 41)		Traditional Iranian Diet (*n* = 52)		*P*-value
	Mean	SD	Mean	SD	
Weight (kg)					
Baseline	83.68	13.49	84.53	13.06	0.760
Day 30	83.41	13.26	84.51	13.51	0.535
Day 60	83.18	13.24	85.75	13.44	**0.024**
Day 90	83.00	13.17	86.37	13.70	**0.001**
*P*-value within groups	0.089		**< 0.001**		
WC (cm)					
Baseline	101.59	7.87	100.96	9.87	0.742
Day 30	99.70	8.56	100.93	10.45	**0.020**
Day 60	99.10	8.51	100.64	10.18	0.073
Day 90	98.32	8.55	100.41	10.81	**0.039**
*P*-value within groups	**< 0.001**		0.161		
BMI (kg/m^2^)					
Baseline	31.08	3.77	31.83	3.64	0.333
Day 30	30.93	3.49	31.93	3.81	0.154
Day 60	30.84	3.42	32.34	3.70	**0.003**
Day 90	30.77	3.47	32.57	3.82	**< 0.001**
*P*-value within groups	**0.032**		**< 0.001**		

*WC* waist circumference, *BMI *Body mass index. *P*-value: Between group comparison (independent t-test for baseline, ANCOVA for other measurement times). *P*-value within groups (RM-ANOVA)

*P* < 0.05 are in bold

In this regard, baseline comparisons using independent t-tests and chi-square analyses indicated no statistically significant differences between the two groups in terms of gender distribution (*P* = 0.726), history of previous weight loss diets (*P* = 0.565), type of school (health-promoting vs. regular; *P* = 0.668), family size (*P* = 0.085), height (*P* = 0.378), or weight (*P* = 0.760). Moreover, the Shapiro–Wilk test was applied to all variables in the study to assess the normality of the distribution of quantitative data. The results indicated that the distribution of all continuous variables was normal.

**Fig. 1:**
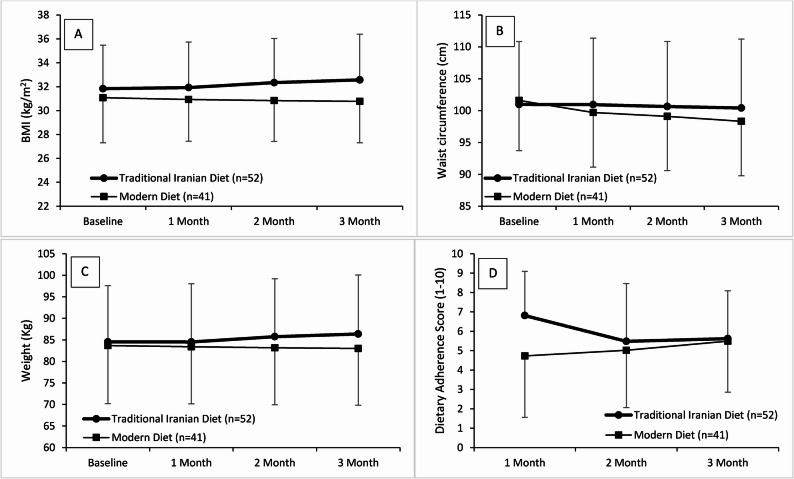
Trends in Weight, BMI, Waist Circumference, and Dietary Adherence Over Time by Diet Group. **A**: BMI changes following 3 months of Modern and Traditional Iranian Dietary interventions; **B**: Waist circumference changes following 3 months of Modern and Traditional Iranian Dietary interventions; **C**: Weight changes following 3 months of Modern and Traditional Iranian Dietary interventions; **D**: Dietary Adherence Score changes following 3 months of Modern and Traditional Iranian Dietary interventions. BMI: Body mass index

Table 1; Fig. 1 illustrate the anthropometric changes during the two dietary interventions. Crucially, the between-group analysis (ANCOVA) revealed statistically significant differences in outcomes. Regarding weight, participants in the modern diet group demonstrated a slight yet consistent reduction over time, though this trend was not statistically significant (*P* = 0.089). In contrast, the traditional Iranian diet group exhibited a statistically significant weight increase by day 90 (*P* < 0.001), with between-group differences becoming significant at day 60 (*P* = 0.024) and day 90 (*P* = 0.001). A similar pattern was observed in BMI, where the modern diet group experienced a modest but statistically significant decrease (*P* = 0.032). Conversely, the traditional Iranian diet group showed a significant increase (*P* < 0.001), resulting in a highly significant between-group difference favoring the modern diet (*P* < 0.001).

On the other hand, WC decreased significantly in the modern diet group (*P* < 0.001), while changes in the traditional Iranian diet group were not statistically significant (*P* = 0.161). Between-group comparisons confirmed the superiority of the Modern Diet, showing significant differences at day 30 (*P* = 0.020) and day 90 (*P* = 0.039) (Table 1; Fig. 1).

According to Table 2, dietary adherence score improved modestly in the modern diet (*P* = 0.033). However, this trend was reversed in the traditional Iranian diet group (*P* = 0.001), leading to a significant between-group difference in adherence by the end of the study (*P* < 0.001). Dietary feasibility was consistently rated higher in the modern diet group across all time points (*P* < 0.001). While this index showed a significant increase for the modern diet during the intervention (*P* = 0.009), it exhibited a significant decrease for the traditional Iranian diet (*P* < 0.001). Family cooperation scores followed the same trend, with between-group analysis showing significantly higher cooperation in the Modern group (*P* = 0.030).

**Table 2:** Dietary adherence, feasibility, and family cooperation scores across intervention periods

Variable	Modern Diet (*n* = 41)		Traditional Iranian Diet (*n* = 52)		*P*-value
	Mean	SD	Mean	SD	
Dietary Adherence Score (1–10)					
Day 30	4.73	3.17	6.81	2.28	**< 0.001**
Day 60	5.02	2.96	5.48	2.98	0.051
Day 90	5.49	2.63	5.62	2.47	0.056
*P*-value within groups	**0.033**		**0.001**		
Dietary Feasibility Score (1–10)					
Day 30	8.85	1.51	7.54	2.00	**< 0.001**
Day 60	9.07	1.40	6.58	2.82	**< 0.001**
Day 90	9.20	1.40	6.25	2.45	**< 0.001**
*P*-value within groups	**0.009**		**< 0.001**		
Family Cooperation Score (1–10)					
Day 30	7.41	2.98	8.46	2.43	0.065
Day 60	7.68	3.03	7.77	3.07	**0.008**
Day 90	7.68	2.94	7.69	3.23	**0.017**
*P*-value within groups	**0.021**		**0.022**		

*P*-value: Between group comparison (independent t-test for baseline, ANCOVA for other measurement times). *P*-value within groups (RM-ANOVA)

*P* < 0.05 are in bold

Regarding changes in the unhealthy dietary patterns, both groups significantly reduced their consumption of junk food over the 90 days, though the modern diet was significantly more effective (Between-group *P* = 0.015). Fast food consumption and drinking soda decreased significantly only in the modern diet group (*P* < 0.001 and 0.002, respectively). Appetite levels, as measured by the CNAQ, declined significantly in both groups (*P* < 0.001), but notably, there were no significant between-group differences in appetite reduction (Table 3).

**Table 3:** Changes in the unhealthy dietary patterns and appetite levels in response to dietary interventions

Variable	Modern Diet (*n* = 41)		Traditional Iranian Diet (*n* = 52)		*P*-value^1^
	Mean	SD	Mean	SD	
Consumption of junk food (chips, pufs, etc.) per week					
Baseline	2.66	2.25	2.35	2.54	0.538
Day 90	1.51	2.16	1.46	1.72	0.051
*P*-value within groups	**0.015**		**0.006**		
Consumption of fast food (per week)					
Baseline	1.66	2.02	1.06	1.65	0.118
Day 90	0.61	0.95	0.81	1.05	0.118
*P*-value within groups	**< 0.001**		0.304		
Drinking soda/soft drinks (per week)					
Baseline	2.56	2.66	2.13	2.38	0.417
Day 90	1.12	1.72	1.60	1.76	0.105
P-value within groups	**0.002**		0.095		
Appetite Level (According to CNAQ)					
Baseline	30.85	3.52	30.11	3.9	0.351
Day 90	28.6	1.98	28.32	2.5	0.559
*P*-value within groups	**< 0.001**		**< 0.001**		

*CNAQ* Council of Nutrition Appetite Questionnaire. *P*-value^1^: Between group comparison (independent t test for baseline, ANCOVA for day 90). *P*-value within groups (paired t test)

*P* < 0.05 are in bold

Meal-skipping behaviours also showed notable changes (Table 4). Skipping breakfast significantly decreased in the modern diet group (*P* = 0.007), while skipping snacks decreased significantly only in the traditional Iranian diet group (*P* = 0.001). However, regarding physical activity levels, no significant difference was observed between the two groups (*P* = 0.285).

**Table 4:** Meal skipping and physical activity patterns before and after dietary interventions

Variable	Modern Diet (*n* = 41)		Traditional Iranian Diet (*n* = 52)		*P*-value
	*N*	%	*N*	%	
Skipping Breakfast					
Baseline	16	39.0%	10	19.2%	**0.035**
Day 90	5	12.2%	13	25.0%	0.121
*P*-value within groups	**0.007**		0.508		
Skipping Other Meals (Lunch & Dinner)					
Baseline	3	7.3%	7	13.5%	0.341
Day 90	1	2.4%	10	20.4%	**0.010**
*P*-value within groups	0.652		0.375		
Skipping Snacks (Morning, Afternoon & Evening Snacks)					
Baseline	21	51.2%	40	76.9%	**0.010**
Day 90	14	34.1%	21	42.8%	0.399
*P*-value within groups	0.230		**0.001**		
Physical Activity (Sedentary /Inactive)					
Baseline	25	61.0%	33	64.7%	0.713
Day 90	29	70.7%	29	59.2%	0.254
*P*-value within groups	0.344		0.678		

*P*-value: Between group comparison (chi-square test). *P*-value within groups (McNemar test)

*P* < 0.05 are in bold

## Discussion

This study, for the first time, directly compared the effectiveness of a modern dietary regimen with a diet based on traditional Iranian medicine principles for weight loss among Iranian students. Our findings indicate a clear divergence in outcomes between the two dietary approaches. Participants adhering to the modern diet exhibited reductions in body weight, WC, and BMI over the intervention period. In contrast, participants assigned to the diet supervised by an Iranian medicine specialist experienced a significant increase in both body weight and BMI. Although the absolute reduction in BMI in the modern diet group was modest, it holds clinical significance when viewed against the backdrop of adolescent development [28]. In the context of puberty and growth spurts, where the natural physiological trajectory often involves weight gain, the stabilization and slight reduction of BMI in the modern diet group represents a positive deviation from the upward trend observed in the traditional diet group.

Moreover, these results suggest that, at least in the context of this study, the modern dietary approach was more effective in promoting weight loss, while the traditional Iranian medicine diet, as implemented, did not lead to favourable anthropometric changes. However, it is critical to interpret these findings within the context of the study design: the modern group followed a strictly quantitative, energy-restricted protocol, while the traditional group followed a qualitative regimen focused on humoral balance rather than caloric deficit.

The effectiveness of Iranian traditional versus modern diets for weight loss remains inconsistent across studies. While some research supports modern dietary approaches, emphasizing reduced energy density and improved diet quality for obesity management [14, 29], others indicate that Iranian traditional methods can be more effective [12, 21]. Comparatively, research from other countries also yields mixed results. For example, a randomized controlled trial showed that a traditional Korean dietary pattern could be as effective as modern diets for weight reduction [30]. However, systematic reviews on traditional Chinese medicine often report inconclusive or mixed evidence, indicating that the effectiveness of traditional approaches varies significantly depending on the specific diet and its implementation [31, 32].

The observed discrepancy in weight loss outcomes may be largely attributable to variations in public knowledge and information accessibility. A key factor contributing to this difference is the pervasive and readily available information on modern diets compared to the relatively limited public understanding of diets based on traditional medicine principles. Modern dietary guidelines and weight loss strategies are extensively disseminated through various channels, including educational textbooks, mainstream media (television, magazines), and especially contemporary social media platforms [33, 34]. This widespread availability of information, often presented in an accessible and easily digestible format, has likely fostered greater familiarity and adherence to modern dietary approaches among the general public, including students and their families [33, 35].

Conversely, diets rooted in traditional medicine, such as the traditional Iranian diet, are often based on complex concepts like “Mizaj” (temperament) and specific food classifications that require nuanced understanding and often individualized guidance [36, 37]. The public’s knowledge of these intricate principles may be limited, and practical, easy-to-follow resources might be scarce compared to the wealth of information available for modern diets [37, 38]. Therefore, the lack of widespread and clear educational materials on the traditional Iranian diet, coupled with the dominant presence of modern dietary information, likely plays a significant role in the observed differential effectiveness.

However, information accessibility is not the sole driver; differences in dietary structure, cultural adaptability, and individual preferences also played critical roles. The traditional diet relies heavily on energy-dense staples like rice (Chelow) and bread (Sangak); without the strict portion control inherent in the modern diet, this structure can inadvertently promote a caloric surplus. Socio-culturally, the strict traditional rules regarding food combinations (e.g., avoiding fish with yogurt) and specific meal timings often clash with the fast-paced, convenience-oriented lifestyle of modern adolescents, reducing the diet’s feasibility. Furthermore, individual preferences for the high palatability of modern foods may create a psychological barrier to sustaining the more subtle, whole-food-based traditional regimen.

This lack of readily accessible and understandable information about the traditional medicine diet, in contrast to the pervasive availability of modern dietary guidelines, is strikingly reflected in the adherence patterns observed in our study. In the present investigation, we found that although dietary adherence, dietary feasibility, and family cooperation scores for the modern medicine diet increased significantly from before to after the intervention, all of these indicators decreased significantly in the traditional Iranian medicine diet group. These results may suggest that the lack of knowledge among students and their parents about the intricacies and practical application of the traditional Iranian diet was a major barrier. Furthermore, this decline suggests ‘diet fatigue’ driven by the complexity of the intervention. Unlike the modern diet, the traditional protocol required strict adherence to specific rules regarding food combinations and meal timings. While initial enthusiasm may have driven high compliance in the first month (the ‘novelty effect’), the restrictiveness and lack of convenience of these traditional rules likely hindered long-term sustainability compared to the more flexible modern approach.

Our findings align with existing research: nutritional knowledge is key to dietary adherence [39, 40]. For example, a review of studies on university students showed that better nutrition knowledge often leads to healthier eating habits [39]. Similarly, an Iranian study found that adults with a better understanding of modern healthy lifestyle guidelines were more likely to adhere to them, which was linked to a lower risk of metabolic syndrome [41]. Our results highlight the need for specific educational programs and accessible resources to promote traditional diets, as historical knowledge alone isn’t enough for practical adherence today.

Beyond differences in knowledge and adherence, our study further revealed that the modern diet was more effective in fostering healthier eating habits among students, leading to significant reductions in unhealthy food consumption, such as junk food, fast food, and soda. This positive shift in dietary choices, which was observed to be more pronounced in the modern diet group, likely contributed substantially to the observed decreases in weight, WC, and BMI during the intervention. Additionally, adherence to the modern diet was associated with a significant reduction in breakfast skipping, a habit frequently linked to poorer dietary quality and potential weight gain [39, 42]. While both diets showed a slight decrease in overall appetite, the modern diet resulted in a more substantial reduction in students’ appetite during the intervention. Taken together, these behavioural and physiological changes-improved food choices, increased breakfast consumption, and greater appetite control-collectively explain the favourable anthropometric outcomes in the modern diet group and, conversely, the observed weight increase in the traditional Iranian medicine group. Moreover, while poor adherence to the traditional Iranian medicine was a factor, the nature of the diet likely contributed. Traditional staples, as mentioned earlier, are energy-dense. In other words, because the traditional protocol focused on qualitative humoral balance without setting a specific calorie limit, participants may have reached a caloric surplus while consuming traditional staples such as rice and bread, which are naturally energy-dense. Consequently, the observed weight gain in this group should be interpreted as a methodological outcome of an ad libitum implementation in an adolescent population, rather than a definitive lack of efficacy of the traditional diet itself.

These findings align with other research on dietary interventions. Studies consistently demonstrate that interventions promoting healthier food choices, including reduced consumption of processed foods and sugary beverages, are effective strategies for weight management [43]. For instance, a systematic review on school-based interventions indicated that programs focusing on healthy eating and environmental changes were most effective in improving dietary behaviour and anthropometry among young people [44]. Furthermore, the reduction in breakfast skipping observed in our modern diet group is often associated with improved weight outcomes and overall dietary quality in various populations, particularly among children and adolescents [39, 42, 45]. The greater appetite suppression noted in the modern diet group could also be a critical factor, as effective appetite control is fundamental for sustained caloric reduction and weight loss [46].

As noted in the protocol description, the primary objective of the traditional Iranian regimen is often to establish disciplined habits and support gastrointestinal health rather than serving as a strictly hypocaloric weight-loss tool [23]. While it resulted in weight gain in this specific population, its emphasis on whole foods and specific food combinations may offer metabolic or digestive benefits for other populations. Future strategies should consider integrating these holistic principles with modern caloric restriction to optimize both weight management and overall well-being [47].

Despite its novel comparative design, this study has several limitations that warrant consideration. First, the absence of a ‘no-intervention’ control group limits our ability to isolate the specific effects of the dietary interventions from the natural growth patterns of adolescents or the ‘participation effect’. Given that adolescence is a period of rapid growth where weight gain is physiologically expected, the lack of a non-intervention control means we cannot definitively determine the extent to which the weight gain in the Traditional group was due to the diet itself versus natural growth. Second, while participants were blinded to the parallel group’s specific protocol and allocation was concealed using sealed envelopes, a double-blind design was not feasible due to the distinct nature of the interventions. While double-blinding is the theoretical gold standard, it is often impractical in behavioral dietary interventions where the provider must actively teach the specific protocol. Consequently, the intervention providers (Nutritionists vs. Traditional Medicine specialists) were not blinded to the therapy they were administering, which may have introduced an implementation bias through mechanisms such as unconscious enthusiasm or differing levels of engagement during counseling. We recommend that future studies address this limitation by adopting more realistic blinding strategies, such as the blinding of outcome assessors. Moreover, the study’s generalizability may be limited as it was conducted solely among first-grade junior high school students in Fasa, Iran. In addition, the assessment of secondary outcomes including dietary adherence, feasibility, and family cooperation relied on VAS. While practical, these self-reported measures are subjective and susceptible to recall and social desirability biases. We recommend future studies incorporate objective compliance markers, such as food diaries or urinary biomarkers, to enhance objectivity. Finally, the study duration of 90 days, while sufficient to observe initial changes, might not be long enough to assess long-term adherence or sustained weight management outcomes for either dietary approach.

## Conclusion

To the best of our knowledge, this randomized controlled trial is among the first to directly compare the effectiveness of a modern dietary pattern to a diet based on traditional Iranian medicine principles for weight loss among Iranian students. Our findings indicate that the Modern Diet intervention was associated with superior weight management outcomes in this cohort. This observed superiority appears to be largely driven by the intervention’s quantitative caloric restriction and the structured professional supervision provided by a nutritionist. Conversely, the traditional Iranian diet, implemented as an *ad libitum* protocol without specific energy limits, was associated with an increase in body weight and BMI. This outcome should be interpreted as a reflection of the methodological absence of quantitative energy control rather than a definitive lack of efficacy of traditional dietary principles themselves. Additionally, differences in outcomes may potentially be influenced by factors such as public familiarity with modern guidelines and information accessibility, which likely facilitated better adherence in the Modern group. The Modern intervention was also linked to positive shifts in dietary behaviors, including reduced consumption of energy-dense snacks and decreased breakfast skipping. Therefore, while the traditional Iranian diet offered holistic value, future applications for obesity management should likely integrate traditional qualitative principles with modern quantitative caloric restriction to ensure effectiveness. Ultimately, for rapid weight control in adolescents, modern structured interventions appear more effective, highlighting the need for adapted guidelines if traditional approaches are to be implemented in contemporary obesogenic environments.

## Supplementary Information

Supplementary Material 1.

## Data Availability

The data underpinning this study&apos;s findings can be obtained from the corresponding author following a reasonable request.
