# Supplementary material for: Do traditional medicine-based diets lead to greater weight loss than modern diets in overweight and obese students? A randomized controlled trial
Source: BMC Complement Med Ther. 2026 Feb 7;26:92. doi: 10.1186/s12906-026-05289-3 (PMC12977662; doi:10.1186/s12906-026-05289-3)
Supplement: Supplementary file 1 — Supplementary Material 1. [file 12906_2026_5289_MOESM1_ESM.docx]

**Supplementary Figure 1: A CONSORT flow diagram**

**Enrolment:**

Assessed for eligibility (n =93)

**Excluded (n = 0):**

Not meeting inclusion criteria (n= 0)

Declined to participate (n = 0) Other reasons (n = 0)

**Randomized (n = 93)**

**Allocation:**

Allocated to Traditional Diet Group (n = 52)

**Allocation:**

Allocated to Modern Diet Group (n = 41)

**Follow-Up:**

Lost to follow-up (n = 0)

**Follow-Up:**

Lost to follow-up (n = 0)

**Analysis:**

Analysed (n = 52)

**Analysis:**

Analysed (n = 41)

**Supplementary Table 1: Sample Daily Meal Plan and Serving Sizes for the Modern Diet Group**

| **Breakfast:**  - **Bread (Carbohydrate)**: .......... palm^*^-sized piece of Sangak/Barbari bread (whole wheat) or .......... palm-sized piece of Lavash/Taftoon bread (thin flatbread) - **Protein:** .......... matchbox-sized piece of cheese (30 g) or .......... egg(s) or .......... bowl of cooked legumes (Ash, warm beans, lentil stew) - **Vegetables: …….** vegetables (cucumber, tomato, lettuce, etc.) - **Nuts**.......... walnut(s) or .......... pistachio(s) or .......... almond(s) |
| --- |
| **Morning Snack:**  - **Fruit:** .......... serving of fruit - **:** .......... “Soghe Talaei” biscuit (Whole wheat biscuits) or equivalent or .......... crispy biscuit or equivalent - **Nuts**: .......... walnut(s) or .......... almond(s) or .......... pistachio(s) |
| **Lunch:**  - **Carbohydrate:** .......... tablespoons of plain rice or .......... tablespoons of mixed rice or .......... cups of cooked pasta or .......... palm-sized piece of Sangak/Barbari bread or .......... palm-sized piece of Lavash/Taftoon bread - **Protein:** .......... cups of cooked legumes or .......... Chicken leg/thigh or .......... chicken breast or .......... matchbox-sized piece of fish or .......... portion of lean red meat or .......... tablespoons of soy protein - **Stew:** .......... ladles of low-fat stew (e.g., celery, eggplant, or herb stew) - **Vegetables:** …... vegetables (raw like cucumber, lettuce, tomato, salad, cabbage OR cooked like herbs, zucchini, eggplant, celery, etc.) - **Dairy:** .......... cups of yogurt or .......... glass of doogh (yogurt drink) or .......... tablespoons of whey |
| **Afternoon Snack:**  - **Fruit:** .......... serving of fruit - **Carbohydrate:** .......... “Soghe Talaei” biscuit (Whole wheat biscuits) or equivalent or .......... crispy biscuit or equivalent - **Nuts:** .......... walnut(s) or .......... almond(s) or .......... pistachio(s) |
| **Dinner**   - **Carbohydrate:** .......... palm-sized piece of Sangak/Barbari bread or .......... palm-sized piece of Lavash/Taftoon bread or .......... tablespoons of cooked rice or .......... cups of cooked pasta - Protein: .......... cups of cooked legumes or .......... Chicken leg/thigh or .......... chicken breast or .......... matchbox-sized piece of fish or .......... chunk of red meat or .......... grams of any meat or .......... matchbox-sized piece of cheese or .......... egg(s) - **Vegetables:** ……. Salad or cooked vegetables - **Dairy:** .......... cups of yogurt or .......... glass of doogh or .......... tablespoons of whey |
| **Before Bed:**   - **Fruit:** .......... serving of fruit - **Dairy:** .......... glass of milk or .......... cups of yogurt |
| *Note: 1 "Palm" refers to a standard Iranian household measure for flatbread (approx. 30g). |
| **1 "Glass" / "Cup":** Refers to a standard household glass (*Liwan*), equivalent to approximately **240 ml**.* |
| This diet must be adjusted by a nutritionist. In this diet, the nutritionist determines the amount of energy intake based on the amount of calories consumed and after deducting 300 to 500 kilocalories based on the student's weight, determines the meals and snacks. It is worth noting that the meals and snacks will be adjusted based on the student's taste. Therefore, not all the blanks in the table below will be filled in; if there is no number in front of the blank, it means that the student should not consume it. |
| **One serving of fruit equals:** |
| \| **Fruit** \| **Quantity** \| \| --- \| --- \| \| Yellow or Black Plum \| 2 medium \| \| Pomegranate \| Half of a medium \| \| Orange \| 1 medium \| \| Raspberry \| 1 glass \| \| Black Mulberry \| ¾ glass \| \| Strawberry \| 1¼ glass \| \| Persimmon \| 2 medium \| \| Apricot \| 2 medium \| \| Apple \| 1 medium \| \| Peach or Nectarine \| 2 small \| \| Pear \| 1 medium \| \| Cherry \| 12 pieces \| \| Banana \| 1 medium \| \| Watermelon \| 1 glass diced \| \| Tangerine \| 2 small \| \| Date \| 2 pieces \| \| Grapefruit \| Half \| \| Melon or Cantaloupe \| 300 g slice \| \| Kiwi \| 2 small \| \| Fig \| 2 medium \| \| Grapes \| 1 cluster (15 grapes) \| |

**Supplementary Table 2: An Exemplar of Iranian Traditional Medicine Recommendations for Standardized Dietary Regimen**

| **Abstract**  This document outlines a standardized dietary program based on the principles of traditional medicine. The primary objective of this regimen is to establish disciplined and healthy eating habits within a structured framework, rather than to serve as a weight-loss protocol. This dietary intervention is believed to be beneficial for various clinical conditions, particularly gastrointestinal ailments. The standard duration of the protocol is two to four weeks, which may be extended based on a physician's clinical judgment and recommendations. |
| --- |
| **1.0 Description of the Dietary Protocol**  **1.1 Morning Meal (Breakfast)**  The morning meal shall consist of:   - *Sangak* bread (a traditional whole-wheat leavened flatbread). - A preserve selected from the following: Citron (*Citrus medica*), Quince (*Cydonia oblonga*), Apple (*Malus domestica*), Carrot (*Daucus carota*), Parsnip (*Pastinaca sativa*), or Ginger (*Zingiber officinale*). Alternatively, honey or grape molasses may be consumed.   **Provisions for the Morning Meal:**   1. Consumption is restricted to the fruit portion of the preserves, excluding the accompanying syrup. 2. The consumption of tea during breakfast is prohibited. 3. A maximum of 10 grams of bovine butter is permissible, if desired.   **1.2 Midday Meal (Lunch)**  The midday meal comprises *Chelow* (parboiled and steamed rice) served with one of the following:   - Chicken or lamb. - Low-liquid Quince *Tas-Kebab* (a type of slow-cooked stew). - Quince stew. - Apple stew. - Artichoke stew.   **Provisions for the Midday Meal:**   1. It is recommended that the *rice* be prepared as one of the following variants:    - **Rice with Cumin:** Cumin seeds are layered within the rice during the steaming process.    - **Rice with Coriander:** Fresh coriander is added to the rice at the final stage of boiling, allowed to blanch briefly, and then drained and steamed with the rice.    - **Rice with Barberry Rice:** Prepared conventionally.    - **Rice with Carrot:** 0.5 kg of julienned carrots are lightly sautéed. A simple syrup (0.5 cup sugar, 0.5 cup water) is added and reduced until evaporated. The parboiled rice is then layered with the prepared carrots and pre-cooked, shredded chicken, and subsequently steamed. 2. Should meat be desired with the meal, the following preparations are permitted (per person serving): 70-100g of chicken fillet or breast, or 40-60g of lean lamb loin, prepared via grilling (on skewers or a grill rack) or poached with minimal water such that the liquid is fully absorbed. Alternatively, the meat may be semi-cooked and layered in the center of the rice during the steaming process. 3. **Tas-Kebab Preparation:** Chicken or lamb is cooked with sliced onions. Sliced carrots and a small quantity of prunes are then added, followed by sliced quince in the final stage. The resulting stew must have minimal liquid and is served with toasted *Sangak* bread or rice. 4. **Quince Stew:** Prepared similarly to a standard meat stew but omitting potatoes and with minimal liquid. Sliced quince is sautéed in minimal oil, sprinkled with sugar until caramelized, and then placed atop the stew before serving. 5. **Apple Stew:** Prepared in a manner analogous to Quince Stew, with apples substituted for quince. 6. **Artichoke Stew:** Cooked lamb to which chopped cardoon/artichoke thistle is added in the final stages of cooking. 7. **Permissible Seasonings:** Salt, black or green cumin, thyme, saffron, cinnamon, and sumac.   **1.3 Evening Meal (Dinner)**  The evening meal may consist of one of the following three options:   - A replication of the prescribed morning meal. - A replication of the prescribed midday meal. - Apple Faloodeh: Apple Faloodeh is an uncooked therapeutic tonic designed to support digestive health and provide gentle nourishment.   **Apple *Faloodeh* Composition:** One medium red apple (or 2-3 small sweet apples), grated, combined with one tablespoon of sugar and two tablespoons of rosewater.  **Provisions for Apple *Faloodeh*:**   1. For individuals with a "hot" temperament (*Garm-Mazaj*), the preparation may be supplemented with one teaspoon of semi-crushed or ground purslane (*Portulaca oleracea*) seeds. 2. Apple *Faloodeh* may be consumed as a standalone intermediate meal or as a main meal accompanied by *Sangak* bread. 3. The substitution of honey for sugar is the preferred method of sweetening. 4. The addition of willow water distillate (*Araq-e Bidmeshk*) (five tablespoons) can enhance its gustatory profile and restorative properties. 5. In cases of constitutional weakness or anemia, the addition of a small quantity of ground saffron is recommended.   **1.4 Intermediate Meals (Snacks)**  The protocol permits the consumption of two intermediate meals daily: one between the morning and midday meals, and one between the midday and evening meals.  The permissible options for these meals are:   - Apple *Faloodeh* (prepared as described in section 1.3). - Fourteen raw almonds (preferably of Iranian origin), which must be masticated slowly and thoroughly. |
| **2.0 General Directives and Protocol Adherence**   1. The ingestion of water and tea is proscribed from 30 minutes prior to 2 hours subsequent to meals. Consumption outside this time is unrestricted. 2. No beverages other than water or weak tea should be consumed between meals. 3. Mealtimes must be strictly regulated and consistent (e.g., 8:00 AM, 1:00 PM, 8:00 PM). 4. The meal schedule should be adapted to the individual's daily activities, but must remain fixed throughout this period. 5. The evening meal should be scheduled for consumption between 7:00 PM and 8:00 PM. 6. Recumbency and sleep are prohibited for two hours postprandially. 7. The sleep schedule should be regulated, with bedtime occurring between 10:00 PM and a maximum of 11:00 PM. 8. Thorough mastication of food is mandated, with the duration of each main meal lasting approximately 20 minutes. 9. The consumption of fruits and other food items not explicitly listed in this protocol is prohibited for the duration of the regimen. 10. Cessation of eating should occur prior to reaching full satiety (conceptually, five spoons before feeling completely full). 11. In the event of experiencing constitutional weakness or faintness, one of the following interventions may be employed:     - Place a walnut-sized piece of waxy honey in the oral cavity. Assume a resting position with the head lowered onto the forearms. Retain saliva in the mouth without swallowing for 15 minutes. After this period, raise the head, swallow the accumulated saliva, and then chew the waxy honey until the sweetness dissipates. The remaining beeswax should be discarded.     - In the absence of waxy honey, allow one tablespoon of honey to dissolve slowly in the oral cavity.     - Slowly masticate 14 roasted (unsalted and un-acidulated) Iranian almonds. 12. Should constipation occur, it is to be managed in consultation with the treating physician. |
